# Identification and Determination of Flavonoids in Astragali Radix by High Performance Liquid Chromatography Coupled with DAD and ESI-MS Detection

**DOI:** 10.3390/molecules16032293

**Published:** 2011-03-09

**Authors:** Yan-Wen Lv, Wei Hu, Yu-Ling Wang, Lan-Fang Huang, Yun-Biao He, Xian-Zhen Xie

**Affiliations:** 1 Department of Chemistry and Material Engineering, Quzhou College, Quzhou 324000, China; 2 Changde Institute for Drug Control, Changde 415000, China; 3 Department of Medicine and Pharmacy, Changde Vocational Technical College, Changde 415000, China

**Keywords:** HPLC, DAD, ESI-MS, flavonoids, astragali radix

## Abstract

A method for the analysis of flavonoids in Astragali Radix by high-performance liquid chromatography (HPLC) combined with photodiode-array detection (DAD) and an electrospray ionization (ESI) - mass spectrometry (MS) was developed. After the samples were extracted with ethanol, the optimum separation conditions for these analytes were achieved using a gradient elution system and a 2.0 × 150 mm Shimadzu VP-ODS column. Eight flavonoids were identified to exist in Astragali Radix based on their characteristic UV data and mass spectra. The concentrations of three major components in this herb—ononin, calycosin and formononetin—were determined by LC/ESI-MS in positive selective ion monitoring (SIM) mode. The calibration curves were linear in the range of 0.9~180.0 μg·mL^−1^ for ononin, 1.8~360.0 μg·mL^−1^ for calycosin and 1.4~280 μg·mL^−1^ for formononetin, respectively. The limits of quantification (LOQ) and detection (LOD) were 0.9 μg· mL^−1^ and 0.2 μg mL^−1^ for ononin, 1.8 μg mL^−1^ and 0.5 μg·mL^−1^ for calycosin, 1.4 μg mL^−1^ and 0.5 μg·mL^−1^ for formononetin, respectively. The standard recoveries were between 95.4~104.7%. The developed method was proven to be useful for the quantitative and qualitative analysis of flavonoid constituents in various resources of Astragali Radix.

## Introduction

Astragali Radix, the dry roots of *Astragalus mongholicus* Bge. [A. membranaceus Bge. var. mongholicus (Bge.) Hsiao] or *A. membranaceus* (Fisch.) Bge. (Leguminosae), known as Huangqi in China, is a Traditional Chinese Medicine (TCM) and one of the most widely used Chinese herbs prescribed in many Chinese formulas to reinforce vital energy. It has many biological functions, such as hepatoprotective, antioxidative, antiviral, antihypertensive [1], so it is frequently used by Chinese doctors as a tonic, an antiperspirant, a diuretic and for treatment of nephritis and diabetes. Triterpene saponins, flavonoids, polysaccharides and biogenic amines are the known constituents found in Astragali Radix are associated with these properties [2,3,4,5,6]. Among them, flavonoids play an important role in human nutrition as health promoting natural chemicals and are established to be the beneficial components. The major flavonoids are formononetin, ononin, calycosin and its glycoside, which are iso-flavonoids. Sometimes 9-methoxynissolin-3-*O*-β-D-glucoside, isomucronulatol-7-*O*-β-D-glucoside, 9-methoxynissolin and isomucronulatol are found in different sources of Astragali Radix. When quality evaluation or standardization of Radix astragali and its products is conducted, the inherent isoflavonoids are chosen as “marker compounds” due to their suitable chromophores for UV detection [7]. Natural products are a major resource in pharmaceutical industry. The research, which looks for guiding compounds for the possible new medicines, is very important. Because the separation process for active components from pharmaceutical plants is very time-consuming with the traditional methods, it is very important to develop high effective, fast, sensitive and selective methods for the analysis of active components in natural plants. For example, recently a high-throughput method for isolation, purification, and characterization of bioactive natural products, which combined many advanced technologies, was reported [8]. Due to these reasons, analytical methods for the analysis of isoflavonoids in Astragali Radix and its products are required in order to support the steady progress of medicinal sciences. There are several techniques for the analysis of isoflavonoids in Astragali Radix and its products, including spectrophotometry [9], high-performance liquid chromatography [10,11] and LC/MS-MS [12]. Reversed-phase HPLC is now commonly used for the separation of complex mixtures of flavonoids in Astragali Radix. However, usually only a limited number of flavonoid compounds have been analyzed due to the often-occurred low resolutions between flavonoids and low UV detection sensitivity. In this paper, a method for simultaneous separation, identification of iso-flavonoids mentioned above in Astragali Radix by HPLC coupled with DAD and mass spectrometric detection was developed. Then, quantitative method for the determination of the three main isoflavonoids, formononetin, ononin and calycosin was validated.

## Results and Discussion

### HPLC-DAD analysis of flavonoid standards

**Figure 1:**
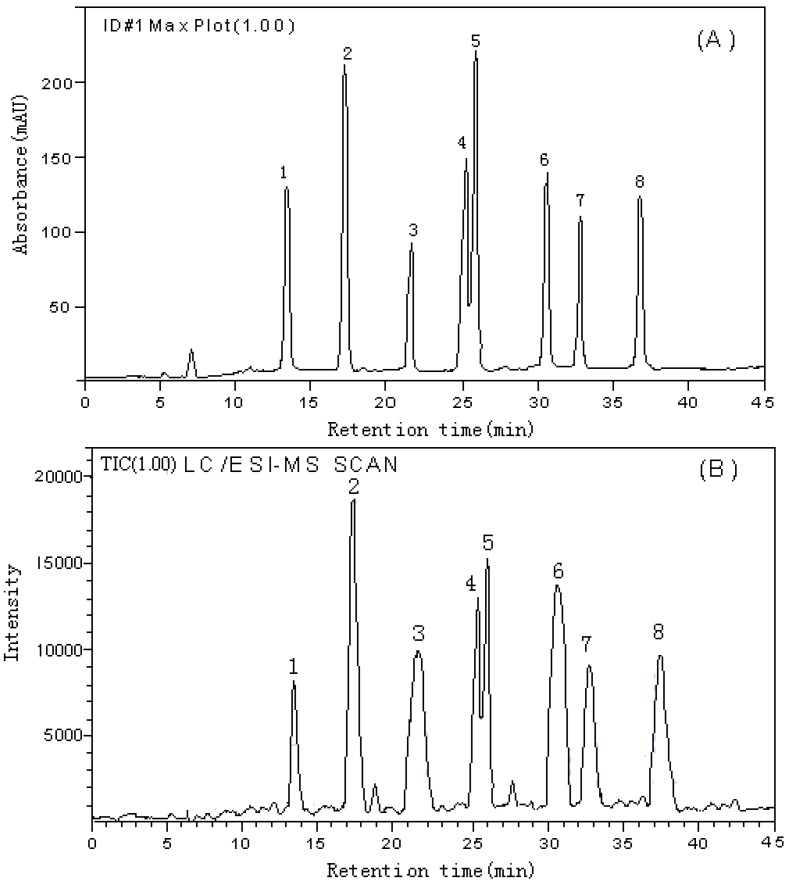
Simultaneous HPLC chromatogram detected with a diode array detector set at 260 nm (A) and LC/ESI-MS total ion chromatograms (TIC) in scan mode (B) of a reference solution of Astragali Radix: (1) calycosin-7-*O*-β-D-glucoside; (2) ononin; (3) 9-methoxynissolin-3-*O*-β-D-glucoside; (4)calycosin; (5) isomucronulatol-7-*O*-β-D-glucoside; (6) formononetin; (7) 9-methoxynissolin; (8) isomucronulatol.

The purpose of this assay was to obtain chromatograms and LC/ESI-MS TIC with better resolution of adjacent peaks within a short analysis time, especially when numerous samples were to be analyzed. The separation of iso-flavonoids was very difficult due to their similar structure. Then, optimizing the chromatographic conditions was essential. Separation conditions such as percentage of water, acetonitrile, acetic acid and solvent elution program were optimized with the reference solution. The optimum chromatographic conditions were summarized in Section “Chromatographic conditions”. As expected, the HPLC-DAD profile was monitored at 260 nm (see Figure 1A). Eight iso-flavonoid standards were separated with good resolution in the optimum chromatographic conditions. The retention time (t_R_) and UV data of each component was summarized in Table 1.

**Table 1:** The values of t_R_, UVλ_max_, [M+H]^+^ and [M+Na]^+^ of flavonoid standard compounds.

Peak No.	Compound name	t_R_	λ_max_	[M+H]^+^	[M+Na]^+^	Fragment ion
		(min)	(nm)	(m/z)	(m/z)	(m/z)
1	Calycosin-7-*O*-β-D-glucoside	13.64	260, 290 sh	447	469	285
2	Ononin	17.21	255, 301 sh	431	453	269
3	9-Methoxy-nissolin-3-*O*-β-D-glucoside	21.49	282	463	485	301
4	Calycosin	25.48	250, 290	285	307	-
5	Isomucronulatol-7-*O*-β-D-glucoside	25.89	282	465	487	303
6	Formononetin	30.73	252, 305	269	291	-
7	9-Methoxynissolin	32.92	285	301	323	-
8	Isomucronulatol	37.08	282	303	335	-

### LC/ESI-MS analysis of flavonoid standards

On-line molecular mass information in the analysis of flavonoid standards was provided by the use of LC/ESI-MS. Atmospheric pressure chemical ionization (APCI) or electrospray ionization (ESI) are soft ionization techniques, which form mainly M^+^ peaks. These M^+^ peaks gave rapid information on the molecular mass of a component directly after its elution from the LC column. In order to obtain optimum ionizing conditions, using the reference solution, both an atmospheric pressure chemical ionization (APCI) and electrospray ionization (ESI) interface were tested in positive and negative ion mode by scanning between m/z 200~550 per second. ESI interface and positive ion mode were chosen. LC/ESI-MS total ion chromatograms (TIC) of a reference solution of Astragali Radix in scan mode were shown in Figure 1B. The optimum ESI-MS conditions were summarized in the section on “Mass spectrometric detection conditions”. Under the optimal chromatographic conditions and ESI-MS conditions, the MS data were obtained, which are also listed in Table 1. There were fragment ions in MS data of calycosin-7-*O*-β-D-glucoside, ononin, 9-methoxynissolin-3-*O*-β-D-glucoside and isomucronulatol-7-*O*-β-D-glucoside, which were consistent with their structure and arising from removal of the related glycoside. This was also helpful for the identification of flavonoids in Astragali Radix extract.

### Analysis of the extract of the roots of A. mongholicus

**Figure 2:**
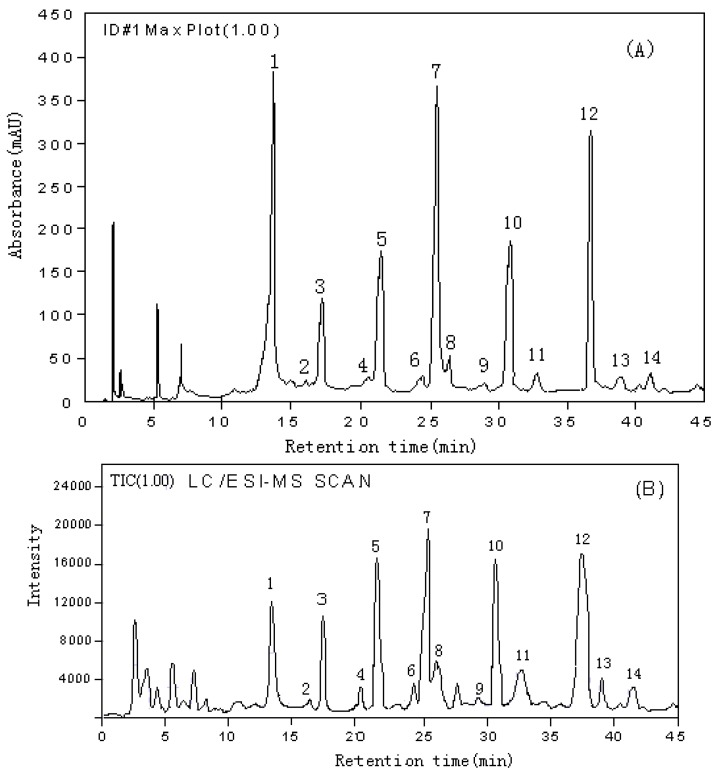
Simultaneous HPLC chromatogram detected with a diode array detector set at 260nm (A) and LC/ESI-MS total ion chromatograms (TIC) in scan mode (B) of A. mongholicus. Peak identification was listed in Table 2.

As shown in Figure 1 and Table 1, each flavonoid showed significant and distinctive [M+H]^+^ or [M+Na]^+^ions, UV spectra, and quite different retention times, consequently, these characteristics of standards can be used for comparison with the chromatograms of various sources of Astragali Radix extracts. As an example, the HPLC-DAD chromatogram detected with DAD set at 260 nm and LC/ESI-MS TIC of extract of *A. mongholicus* from Shanxi in scan mode were shown in Figure 2. As shown in Figure 2A, there were fourteen major peaks (1~14) in the retention range from 10 to 45 min and their UV spectra were obtained using the photodiode array detector during HPLC analysis. Eight of these fourteen peaks could be correlated to isoflavonoids from the UV spectra by two absorbance bands around 258 and 288 nm. However, due to the low selectivity and sensitivity of assignment by UV spectra, it was difficult to identify each component, so the MS spectra of the fourteen peaks were also measured using LC/ESI–MS in scan mode (see Figure 2B). MS data of each identified peak together with its retention time and UV spectrum data were shown in Table 2. Peak assignment was also listed in Table 2.

**Table 2:** Peak assignment of A. mongholicus extract.

Peak No.	t_R_ (min)	λ_max_ (nm)	[M+H]^+^ (m/z)	[M+Na]^+^ (m/z)	Fragmention (m/z)	Identification
1	13.65	260, 290sh	447	469	285	Calycosin-7-O-β-D-glucoside
3	17.22	255, 301sh	431	453	269	Ononin
5	21.47	282	463	485	301	9-Methoxy-nissolin-3- O-β-D-glucoside
7	25.48	250, 290	285	307	-	Calycosin
8	25.89	282	465	487	303	Isomucronulatol-7-O-β-D-glucoside
10	30.71	252, 305	269	291	-	Formononetin
11	32.94	285	301	323	-	9-Methoxy-nissolin
12	37.09	282	303	335	-	Isomucronulatol

The same procedure was used for study on the roots of *A. membranaceus*. The results showed that both species contained nearly the same flavonoids as described above. However, the flavonoid content of the extract was much lower than that of *A. mongholicus*. This was consistent with an earlier report [12].

### Validation: calibration curves, linearity and detection limit

**Figure 3:**
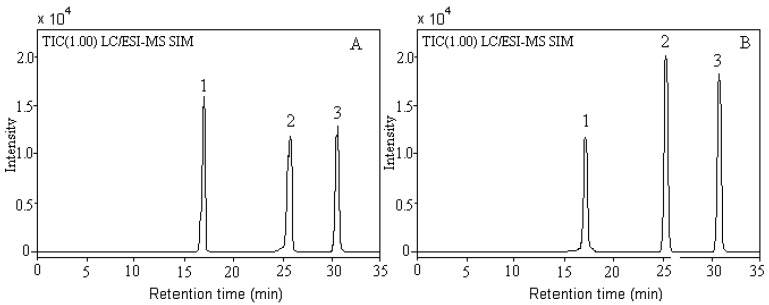
LC/ESI-MS total ion chromatograms (TIC) of Astragali Radix flavonoid standards containing 60.0 μg·mL^−1^ for ononin, 40.0 μg·mL^−1^ for calycosin and formononetin (A) and *A. mongholicus* (B). Peaks: (1) ononin; (2) calycosin; (3) formononetin.

Formononetin, ononin and calycosin were the most important biologically active components among the isoflavonoids of Astragali Radix, therefore, quantitative determination of these three isoflavonoids is essential for quality control of this commonly used drug [13,14]. The LC/ESI-MS TIC of mixed standards of formononetin, ononin and calycosin in positive SIM mode is shown in Figure 3A. The calibration curves were linear in the range of 0.9~180.0 μg·mL^−1^ for ononin, 1.8~360.0 μg·mL^−1^ for calycosin and 1.4~280 μg·mL^−1^ for formononetin, respectively. The calibration curves were Y = 1673X + 1142, Y = 1428X + 1473 and Y = 1561X + 1326 with correlation coefficients of 0.9984, 0.9975, and 0.9985 for ononin, calycosin and formononetin, respectively. Because Astragali Radix contained analyte, no real blank was available for preparation of standards or controls. A solvent blank was analyzed for determining limit of detection (LOD) and no peaks at m/z 431 (ononin), m/z285 (calycosin) and m/z 269 (formononetin) were observed in the blank. The limits of detection were 0.2 μg·mL^−1^ for ononin, 0.5 μg·mL^−1^ for calycosin and formononetin, which determined from signal-to-noise ratio of 3:1. The lower limit of the range of the calibration curve can be considered the limits of quantification of this method. The limits of quantification of this method was 0.9 μg·mL^−1^ for ononin, 1.8 μg·mL^−1^ for calycosin and 1.4 μg·mL^−1^ for formononetin.

### Validation: precision, accuracy and recovery

Due to the use of SIM mode in this method, no interference at the retention times of ononin, calycosin and formononetin was observed. Under these conditions, the reported method showed favorable selectivity. The relative standard deviations of retention times of ononin, calycosin and formononetin were 0.24, 0.18 and 0.13, respectively. Six replicate analyses of quality control samples and a spiked sample were used to calculate the precision and accuracy. The results were listed in Table 3. The accuracy was in the order of −2.0–5.5% for ononin, −3.5–3.0% for calycosin and −2.6–4.0% for formononetin. The precision was in the order of 1.6–6.4% for ononin, 1.4–5.8% for calycosin and 1.5–6.1% for formononetin. Various concentrations of standard solutions of ononin, calycosin and formononetin were added to 2.0 g Astragali Radix and recoveries were determined using the conditions described above. The results were shown in Table 4. The recoveries were between 95.4~104.7%. As showed in Table 3 and Table 4, recovery, precision and accuracy of the developed method were very satisfactory. Obviously, the developed method has a very wide linear range, good selectivity, low limits of detection and quantification.

**Table 3:** Summary of precision and accuracy of ononin, calycosin and formononetin.

Analyte	Concentration added	Concentration found	RE	RSD
	(μg•mL^−1^)	(μg•mL^−1^)	(%)	(%, n = 6)
Ononin	2.0	2.11	5.5	6.4
	20.0	19.6	−2.0	3.2
	30.0^a^	30.8	2.7	1.8
	140.0	141.5	1.1	1.6
Calycosin	2.0	1.93	−3.5	5.8
	20.0	20.6	3.0	3.4
	80.0^a^	78.4	−2.0	1.7
	140.0	138.2	−1.3	1.4
Formononetin	2.0	2.08	4.0	6.1
	20.0	20.5	2.5	3.7
	50.0 ^a^	48.7	−2.6	2.4
	140.0	142.1	1.7	1.5

^a^ Water extract of *A.* *mongholicus* spiked with ononin, calycosin and formononetin standards.

**Table 4:** Determination results of the three active components in A. mongholicus and A. membranaceus.

Samples^a^	Components	Found^b^	Added	Increased	Recovery
		(μg•g^-1^)	(μg•g^-1^)	(μg•g^-1^)	(%,)
A	Ononin	41.2	40 50	41.7 48.6	104.3 97.2
	Calycosin	80.6	70 90	68.3 88.4	97.6 98.2
	Formononetin	67.5	50 70	50.8 68.7	101.6 98.1
B	Ononin	32.4	30 40	31.4 39.1	104.7 97.8
	Calycosin	65.7	50 70	47.7 68.2	95.4 97.4
	Formononetin	46.8	40 50	39.2 51.4	98.0 102.8

^a^ A is *A. mongholicus* from Neimonggol and B is *A. membranaceus*; ^b^ Mean value of five replicate analyses.

### Application

The developed method has been applied to quantitative determination of ononin, calycosin and formononetin in Astragali Radix. The typical TIC of extract of *A. mongholicus* from Neimonggol is shown in Figure 3B. As examples, the analytical results of two typical samples are summarized in Table 4. The concentrations of ononin, calycosin and formononetin in Astragali Radix from many other sources were also determined and compared (results were not listed in this paper). The concentrations of these three main isoflavonoids in *A. mongholicus* and *A. membranaceus* were obviously higher than other kinds of Astragali Radix. The quantity of major components can be different even for the same kind of Astragali Radix when Astragali Radix was obtained from different locations. Since isoflavonoids are important biologically active components, quality control of this commonly used drug is therefore a necessity. Determination of the three principal isoflavonoids can be of great importance for the identification, differentiation and quality evaluation of Astragali Radix samples.

## Experimental

### Apparatus

All experiments were carried out on a Shimadzu LCMS-2010 instrument (Kyoto, Japan), which included a LC-10Advp solvent delivery pump, a FCV-10ALvp low pressure gradient unit, a DGU-14A degasser, a CTO-10Avp column oven, a SPD-M10Avp photodiode array detector, and a quadrupole mass spectrometer.

### Chromatographic conditions

The column utilized for separation was a 2.0 × 150 mm Shimadzu VP-ODS column with a particle size of 5 µm. The analytical column was protected by a C_18_ Guard-Pak cartridge (Waters, Milford, MA, USA). The mobile phase consisted of (A) distilled water containing 0.2% (v/v) acetic acid and (B) acetonitrile containing 0.2% (v/v) acetic acid. Each component of the mobile phase was filtered through a 0.22 μm membrane. All separations were at room temperature and a flow-rate of 0.2 mL/min. A gradient program was adopted as follows: linear from 25 to 32% B (0–5 min), linear from 32 to 35% B (6–10 min), linear from 35 to 60% B (11–20 min), and then held there for 25 min. The injection size was 5 μL. The HPLC-DAD chromatographic profile was recorded at 260 nm.

### Mass spectrometric detection conditions

The ESI-MS spectra were acquired in the positive ion and scan mode using an electrospray interface. The positive selective ion monitoring (SIM) mode and [M+H]^+^ at m/z 431, 285, 269 which was selected as the SIM ion, were chosen for quantification of the three main isoflavonoids: formononetin, ononin and calycosin. The ionization parameters for both scan and SIM mode were as follows. ESI temperature was 350 °C. Curved desolvation line (CDL) and block temperature were 240 °C and 210 °C, respectively. Probe voltage was +4.5 KV. Detector voltage was 1.8 kV. CDL Voltage was −16 V. Q-array Bios was 42 V. Nebulizing gas flow was 4.5 L/min.

### Materials

The plant materials used in this study was purchased from a local drug store (Jiu Zhi Tang Pharmacy, Changsha, China) and they were identified to be the dry roots of *A. mongholicus* and *A. membranaceus* by a researcher from Institute of Herbal Drugs, Hunan Academy of Traditional Chinese Medicine. Standards of calycosin-7-*O*-β-D-glucoside (**1**), ononin (**2**), 9-methoxynissolin-3-*O*-β-D-glucoside (**3**), calycosin (**4**), isomucronulatol-7-*O*-β-D-glucoside (**5**), formononetin (**6**), 9-methoxy-nissolin (**7**) and isomucronulatol (**8**) were graciously provided by the Hunan Institute of Chinese Herbal Drugs. A reference solution (10 mL) was prepared with methanol, which contained **1** (0.4 mg), **2** (0.8 mg), **3** (0.8 mg), **4** (0.4 mg), **5** (0.4 mg), **6** (0.4 mg), **7** (0.4 mg) and **8** (0.4 mg). One mg·mL^−1^stock solutions of formononetin, ononin and calycosin were prepared in methanol. A mixed working solution containing 180.0 μg·mL^−1^ ononin, 360.0 μg·mL^−1^ calycosin and 280 μg·mL^−1^ formononetin was prepared by diluting above stock solutions with methanol. By serial dilution of the working solution with methanol, calibration standards at levels of 180.0, 90.0, 18.0, 9.0, 4.5, 1.8 and 0.9 μg·mL^−1^ for ononin, 360.0, 180.0, 36.0, 18.0, 9.0, 3.6 and 1.8 μg·mL^−1^ for calycosin, 280.0, 140.0, 28.0, 14.0, 7.0, 2.8 and 1.4 μg·mL^−1^ for formononetin, were obtained. All the stock solutions and working solutions were stored in a refrigerator (at 4 °C) and brought to room temperature before use. Methanol and acetonitrile were of chromatographic grade. Other regents were of analytical grade. Each component of the mobile phase was filtered through a 0.45 μm membrane.

### Sample preparation

After *A. mongholicus* powder or *A. membranaceus* (2.0 g) was refluxed with methanol (200 mL) for 3 hours, the raw methanolic extract was filtered and the filtrate was evaporated to dryness under vacuum. The viscous residue was swirled in hot water (25 mL) and the suspension was extracted twice with water saturated *n*-butanol (10 mL and 5 mL) in a separatory funnel. The *n*-butanol phases were combined, and *n*-butanol was evaporated to dryness under vacuum. The residue was dissolved in methanol (2.0 mL). An aliquot (5 μL) of the sample solution filtered with a 0.25 μm membrane was injected into the HPLC column.

### Validation of quantitative method

Each calibration standard was determined three times. The standard curves, which were calculated by plotting the peak area ratio (Y) of each analyte in the total ion chromatogram (TIC) of the LC/ESI-MS versus concentration (X, μg·mL^−1^) with least squares linear regression. The retention times of ononin, calycosin and formononetin in LC/ESI-MS TIC were 17.21, 25.48 and 30.73 min, respectively. The method has been validated for selectivity, linearity, precision, accuracy and recovery. Quality control samples of each analyte at concentrations of 2.0, 20.0 and 140 μg·mL^−1^ were prepared by diluting the stock solution with the mobile phase. The precision and accuracy were determined by six replicate analyses of quality control samples. 60.0 μg ononin, 160.0 μg calycosin and 100.0 μg formononetin at sample level were added to *A. mongholicus* powder and performed as indicated under Sample preparation. This spiked sample was also used to evaluate precision and accuracy. Various standard concentrations at sample level of ononin, calycosin and formononetin (see Table 4) were added to 2.0 g actual samples, after extracting as described under Sample preparation, the concentration of these components was determined and recoveries were calculated. The recovery was evaluated by comparing the peak area response of analytes in extracted samples and standard added samples.

## Conclusions

A high-performance liquid chromatography (HPLC) combined with DAD and ESI-MS method for the identification of isoflavonoids in Astragali Radix was developed. As examples, eight isoflavonoids were identified in *A. mongholicus* and *A. membranaceus*, two of the most important Astragali Radix species. With positive SIM mode, a LC/ESI-MS method was validated for quantitative determination of three most important isoflavonoids (ononin, calycosin and formononetin) in Astragali Radix with acceptable precision, accuracy, recovery, selectivity and sensitivity. The developed method can be used for differentiation and quality evaluation of Astragali Radix from different sources.
